# Infection and Immunometabolism in the Central Nervous System: A Possible Mechanistic Link Between Metabolic Imbalance and Dementia

**DOI:** 10.3389/fncel.2021.765217

**Published:** 2021-11-02

**Authors:** Noriko Shinjyo, Kiyoshi Kita

**Affiliations:** ^1^School of Tropical Medicine and Global Health, Nagasaki University, Nagasaki, Japan; ^2^Laboratory of Immune Homeostasis, WPI Immunology Frontier Research Center, Osaka University, Suita, Japan; ^3^Department of Host-Defense Biochemistry, Institute of Tropical Medicine, Nagasaki University, Nagasaki, Japan

**Keywords:** infection, immunometabolism, glia, neuroinflammation, dementia, leptin, insulin

## Abstract

Metabolic syndromes are frequently associated with dementia, suggesting that the dysregulation of energy metabolism can increase the risk of neurodegeneration and cognitive impairment. In addition, growing evidence suggests the link between infections and brain disorders, including Alzheimer’s disease. The immune system and energy metabolism are in an intricate relationship. Infection triggers immune responses, which are accompanied by imbalance in cellular and organismal energy metabolism, while metabolic disorders can lead to immune dysregulation and higher infection susceptibility. In the brain, the activities of brain-resident immune cells, including microglia, are associated with their metabolic signatures, which may be affected by central nervous system (CNS) infection. Conversely, metabolic dysregulation can compromise innate immunity in the brain, leading to enhanced CNS infection susceptibility. Thus, infection and metabolic imbalance can be intertwined to each other in the etiology of brain disorders, including dementia. Insulin and leptin play pivotal roles in the regulation of immunometabolism in the CNS and periphery, and dysfunction of these signaling pathways are associated with cognitive impairment. Meanwhile, infectious complications are often comorbid with diabetes and obesity, which are characterized by insulin resistance and leptin signaling deficiency. Examples include human immunodeficiency virus (HIV) infection and periodontal disease caused by an oral pathogen *Porphyromonas gingivalis*. This review explores potential interactions between infectious agents and insulin and leptin signaling pathways, and discuss possible mechanisms underlying the relationship between infection, metabolic dysregulation, and brain disorders, particularly focusing on the roles of insulin and leptin.

## Introduction

Dementia is a general term for debilitating conditions, in which progressive and long-lasting loss of mental ability impairs cognition and simple daily activities. Alzheimer’s disease (AD), the most common form of dementia, is a neurodegenerative disorder characterized by cognitive decline associated with the accumulation of β-amyloid (Aβ) plaques and neurofibrillary tangles in the brain (Ferri et al., 2005). Amyloid cascade hypothesis is a model postulating a linear pathway initiated by Aβ deposition, eventually leading to neuroinflammation and neuronal loss. Although amyloid cascade hypothesis has provided the theoretical framework for the research direction and advanced the knowledge and understanding of AD pathology at molecular levels in the last decades, it remains controversial whether Aβ is the cause of the pathogenesis (De Strooper and Karran, 2016).

While the exact role for Aβ in dementia etiology remains unclear, several elements have been suggested to increase the risk of cognitive decline. Among those suggested are impaired glycemic control [e.g., metabolic syndromes (MetS) including diabetes] and infection (Ott et al., 1999; Peila et al., 2002; Arvanitakis et al., 2004; Biessels et al., 2006; Irie et al., 2008; Biessels and Despa, 2018). Largely due to modern lifestyle and diet (i.e., the lack of exercise and food with high sugar and carbohydrate), overweight, obesity, and associated metabolic disorders are widespread epidemics. Obesity is a common risk factor for many chronic disorders, such as type 2 diabetes (T2DM) and cardiovascular diseases (Haslam and James, 2005). In addition, evidence suggests that metabolic disturbance can cause neurodegenerative disorders, including AD (Whitmer et al., 2005; Kivipelto et al., 2006; Mejido et al., 2020), possibly via blood–brain barrier (BBB) disruption and neuroinflammation (Pugazhenthi et al., 2017). The association may also be compounded and influenced by other factors, such as age (Bos et al., 2017; Deckers et al., 2017; Whitmer et al., 2018), indicating the complexity of mechanisms underlying the link between metabolic disturbances and cognitive decline. Meanwhile, the relationship between infections and dementia, especially late-onset AD, has been repeatedly suggested over the past three decades (Ashraf et al., 2019). The aim of this review is to overview and discuss how these two contributory causes may be intertwined in the etiology of neurodegenerative disorders, particularly focusing on the interaction between infectious agents and two metabolic hormones, namely insulin and leptin, in the brain and periphery.

## Metabolic Disturbance and Cognitive Impairment

### Insulin and Leptin – Linking Metabolic Syndromes, Infection, and Cognitive Impairment

**FIGURE 1:**
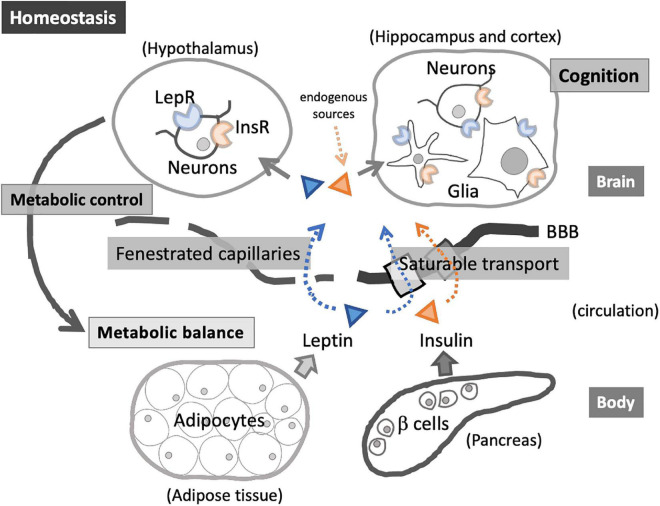
Insulin and leptin in homeostatic interactions between the peripheral organs and central nervous system (CNS). Insulin and leptin enter the brain via saturable transport through blood–brain barrier (BBB) and via fenestrated capillaries in the specific brain regions (e.g., hypothalamus). Acting on InsR and LepR, these hormones exert diverse roles, including energy balance regulation through hypothalamic-pituitary-adrenal axis as well as the modulation of glial immunological activities. InsR and LepR on neuronal cells are involved in cognition.

Energy homeostasis is regulated by intricate interactions between the peripheral organs and central regulatory system in the brain, where insulin and leptin play crucial roles (Boucsein et al., 2021) (Figure 1). Insulin and leptin resistance, as well as dysregulation of related pathways, are associated with obesity and metabolic disorders (Francisco et al., 2019; Gruzdeva et al., 2019) and central nervous system (CNS) dysfunctions (Cereda et al., 2007; Arnold et al., 2018; Kellar and Craft, 2020). Growing evidence suggests that insulin and leptin play significant physiological roles in cognition (Paz-Filho et al., 2008; Morrison, 2009; Gray et al., 2014; Arnold et al., 2018), and these signaling pathways may be promising therapeutic targets to alleviate cognitive impairment accompanied by obesity and MetS (Mejido et al., 2020). On the other hand, obesity, visceral adiposity in particular, is frequently associated with immune dysregulation and infection susceptibility (Hamdy et al., 2006; Conde et al., 2010; Kumari et al., 2019; Obradovic et al., 2021), while infection increases the risk of neurodegeneration and dementia (Heneka et al., 2020; Shinjyo et al., 2021), suggesting the link between MetS, infection, and cognitive impairment.

### Insulin

#### Insulin and Dementia

Insulin is a peptide hormone composed of 51 amino acids, generated from the precursor proinsulin through a series of processing (Rahman et al., 2021). Primarily produced by β cells of the pancreatic islets, insulin is the main anabolic hormone that regulates the energy metabolism throughout the body, i.e., promoting glucose uptake into the liver, fat, and muscle cells (White, 2003). In addition, acting through common receptors (insulin receptor [InsR] and IGF-1 receptor [IGF-1R]), insulin and insulin-like growth factors (IGFs) regulate proliferation and survival of various cell types throughout the body during development, in adulthood, and in aging processes (Nakae et al., 2001; Richardson et al., 2004). InsR and IGF-1R are tyrosine kinase receptors that can be present as homodimers (InsR/InsR, IGF-1R/IGF-1R) or heterodimer (InsR/IGF-1R), sharing the majority of downstream pathways, namely phosphoinositide 3-kinase (PI3K), serine threonine kinase Akt, glycogen synthase kinase 3β (GSK3β), and mammalian target of rapamycin (mTOR) (O’Neill et al., 2012). InsR and IGF-1R are highly expressed in the CNS, including the hippocampus and hypothalamus, and central insulin regulates peripheral energy metabolism (O’Neill et al., 2012; Tiedemann et al., 2017; Barrios et al., 2021; Scherer et al., 2021). Furthermore, it is now evident that insulin and IGF-1 play important roles in higher-order brain functions, such as memory and cognitive processing, and in neuroprotection in response to brain injury (Stewart and Rotwein, 1996; Gerozissis, 2003; Fernandez and Torres-Aleman, 2012), indicating pleiotropic roles for insulin/IGF signaling in the CNS.

Insulin crosses the BBB using a saturable transporter, which is regulated by insulin itself and altered by a number of factors including hyperglycemia and diabetes (Banks et al., 2012). In addition, the choroid plexus, a highly vascularized tissue responsible for the production of cerebrospinal fluid (CSF) at the interface of the CNS and periphery, releases insulin and IGFs (Salehi et al., 2009; Ziegler et al., 2012; Mazucanti et al., 2019; Dani et al., 2021). In the parenchyma, microglia generate IGF-1 during development (Wlodarczyk et al., 2017) as well as in adulthood (Myhre et al., 2019), while astrocytes can produce insulin, which is negatively regulated by Aβ and bacterial lipopolysaccharide (LPS) (Takano et al., 2018). InsR and IGF-1R are expressed on neurons and non-neuronal cells (Shaughness et al., 2020), and are involved in the regulation of synaptic plasticity (Dyer et al., 2016), astroglial energy metabolism, microglial inflammatory phenotypes (Haas et al., 2020), as well as the self-renewal and maintenance of neural stem cells (Ziegler et al., 2015), indicating crucial roles of insulin through multiple targets and mechanisms in the brain parenchyma (Gabbouj et al., 2019). Although the hypothalamus is the most well-studied target region regulating systemic energy metabolism (Mitchell and Begg, 2021), specific inactivation of InsR and IGF-1R in the hippocampus and amygdala led to increased anxiety and cognitive impairment in animal models (Soto et al., 2019), indicating the significance of extrahypothalamic and non-metabolic roles for insulin. Moreover, hippocampal microglia responded to insulin treatment in young rats but not in the aged group (Haas et al., 2020), suggesting that microglia develop insulin resistance during aging. Indeed, experimental evidence suggests the link between impaired insulin signaling in the CNS and cognitive impairment. Intracerebral streptozotocin (STZ) induces AD-like brain pathology in mice, which has been widely used as a model for sporadic AD (Lester-Coll et al., 2006). In mixed AD and diabetic mice using APPswe/PS1dE9 (APP/PS1) transgenic AD model or triple-transgenic model of AD (3xTg-AD) with either STZ treatment or InsR deficiency (*db*/*db*), genetic background and dysfunctional insulin signaling cooperatively exacerbated CNS inflammation and AD pathology (Hierro-Bujalance et al., 2020; Imamura et al., 2020; Sankar et al., 2020). In addition, intranasal insulin treatment can alleviate AD pathogenesis and cognitive impairment via reduced neuroinflammation and enhanced neural plasticity (Chen Y. et al., 2014; Guo et al., 2017), possibly via the actions through receptors on microglia and astrocytes (Spielman et al., 2015). Although what roles insulin/IGF signaling play in the CNS could be context-dependent, i.e., either beneficial (Carro et al., 2003; Sukhanov et al., 2007; Tien et al., 2017) or damaging (Labandeira-Garcia et al., 2017), fine-tuning of these signaling pathways is crucial to maintaining brain functions. Considering the anti-inflammatory effects of insulin and IGF-1 on microglia (Labandeira-Garcia et al., 2017; Shaughness et al., 2020) and observations that age-related changes of microglia toward pro-inflammatory phenotypes are implicated in brain aging and neurodegenerative disorders (Gemma et al., 2010; Chowen and Garcia-Segura, 2020), microglial insulin/IGF resistance may underlie chronic inflammation in the brain, which is associated with dementia (Lutshumba et al., 2021). Consequently, dysregulated insulin/IGF signaling and insulin resistance in the CNS have been linked to an increased risk of dementia, including AD, which is sometimes referred to as type 3 diabetes (Biessels et al., 2006; Whitmer et al., 2008; Zemva and Schubert, 2011; Banks et al., 2012; Smolina et al., 2015; Biessels and Despa, 2018; Kuo et al., 2018; Shinjyo et al., 2020).

#### Insulin as an Immunomodulator – A Possible Link Between Metabolic Syndromes and Infection Susceptibility

Acute and chronic infections induce insulin resistance (Yki-Järvinen et al., 1989; Fernández-Real et al., 2006), whereas metabolic imbalance (e.g., higher body fat mass and diabetes) is associated with higher infection susceptibility (Rayfield et al., 1982; Fernández-Real et al., 2007), indicating bidirectional interactions between the immune system and energy metabolism in human body. Chronic low-grade inflammation and immune dysregulation likely mediate the mutual and possibly synergetic relationship. The strong interaction between the immune and metabolic pathways is apparently rooted in their common evolutionary origin, as represented by the fat body in *Drosophila*, which senses both infectious and metabolic stresses and perform the functions of the liver, adipose tissue, and immune system (Hotamisligil, 2017). Insulin plays a key role in such evolutionarily conserved immunometabolism, partly through the interactions with tumor necrosis factor (TNF) receptor (Uysal et al., 1997) and Toll-like receptor (TLR) pathways (DiAngelo et al., 2009; Hotamisligil, 2017), as well as through modulating metabolic pathways in immune cells (van Niekerk et al., 2020). Through metabolic regulation, insulin alleviates the harmful effects of hyperglycemia (Sun et al., 2014). For example, due to its glucose-lowering effect, insulin exert anti-inflammatory effects through modulating the release of inflammatory mediators. As glucose is pro-inflammatory, insulin deficiency activates inflammatory reactions in the body, leading to the release of inflammatory mediators including reactive oxygen species and pro-inflammatory cytokines from leukocytes (Mohanty et al., 2000; Esposito et al., 2002). In addition, insulin directly activates phagocytic and bactericidal activity of immune cells and diabetes-induced infection susceptibility is partly mediated by impaired immune responses due to the lack of insulin signaling, as shown in rodent studies (Yano et al., 2012).

### Leptin

#### Leptin as a Metabolic Hormone

Leptin is a 16 kDa polypeptide that regulates metabolic balance and fat storage. Mainly produced by the white adipose tissue (WAT), leptin acts via leptin receptor (LepR) in the brain and plays a pivotal role in the control of appetite and energy expenditure. There are six LepRs (LepRa to LepRf), with identical extracellular N-terminal domain and distinct intracellular C-terminal regions generated by alternative splicing of *db* (Lee et al., 1996). LepRb, the long isoform with high ligand affinity, is the major isoform expressed in the brain and activates intracellular pathways, including JAK/STAT, ERK/MAPK, and IRS/PI3K (Allison and Myers, 2014). The hypothalamic nuclei, the regulatory center of energy homeostasis, are highly enriched with LepRb (Elmquist et al., 1998; Balthasar et al., 2004; Leshan et al., 2009), and leptin exerts its effects through the action in the hypothalamus to regulate food intake and energy metabolism (Friedman, 2019). Leptin enters the hypothalamus through fenestrated capillary and acts on LepR expressed on neurons in arcuate nucleus (ARC), dorsomedial hypothalamus (DMH), and ventromedial hypothalamus (VMH), enabling a feedback mechanism to maintain energy balance, thereby preventing obesity and metabolic disorders (Pandit et al., 2017). In addition, leptin can cross BBB using a saturable transport system (Banks and Farrell, 2003), as well as the blood-CSF barrier (choroid plexus epithelia) (Merino et al., 2006; Dietrich et al., 2008). LepRb in extrahypothalamic brain regions also plays significant roles in the regulation of energy metabolism (Scott et al., 2009). For example, LepRb in the ventral tegmental area (VTA) regulates energy balance via mesolimbic dopaminergic system (Fulton et al., 2006; Hommel et al., 2006), suggesting that leptin targets multiple brain regions and cellular components. Consequently, dysregulation of leptin signaling results in obesity, diabetes, and associated comorbidities (Myers et al., 2008; Wasim et al., 2016; Fischer et al., 2020). Leptin resistance is the condition where diminished leptin sensitivity occurs, resulting in a defect in satiety detection despite high leptin levels, which has been linked to obesity (Izquierdo et al., 2019). Leptin deficient (*ob*/*ob*) and LepR deficient (*db*/*db*) mice, carrying mutations in leptin (*ob*) and LepR (*db*) genes, respectively, exhibit excessive eating, develop obesity and diabetes, and are widely used as animal models of T2DM (Chen et al., 1996; Ninomiya et al., 2002; Gautron and Elmquist, 2011). Growing evidence suggests the contribution of leptin resistance to neurodegeneration in AD (Bonda et al., 2014). Serum leptin levels showed negative correlation with cognitive decline in the elderly (Holden et al., 2009). In the brain autopsy, CSF leptin levels were significantly higher in AD compared to control and mild cognitive impairment cases, and CSF leptin concentration was correlated with pathological neurofibrillary tangle burden (Bonda et al., 2014), suggesting that leptin resistance develops during AD progression.

#### Leptin as an Adipokine

Adipokines are the adipose tissue-derived factors that affect whole body homeostasis in autocrine and paracrine manners, targeting a number of biological processes such as glucose metabolism, lipid metabolism, insulin sensitivity, as well as immune response (Fasshauer and Blüher, 2015). Adipokines include leptin, adiponectin, vaspin, fibroblast growth factor 21 (FGF21), and many more, each exerting specific biological effects, and mediate diverse actions throughout the body (Fasshauer and Blüher, 2015). Originally identified as an adipocyte-derived hormone that regulates neuroendocrine axis, leptin is one of the most studied adipokines linking the immune system and energy metabolism (Abella et al., 2017; Jiménez-Cortegana et al., 2021).

Indeed, leptin belongs to the family of long-chain helical cytokines and has similarity to IL-6, IL-12, and granulocyte colony-stimulating factor (G-CSF). LepR is a type I cytokine receptor (La Cava and Matarese, 2004). Although neurons are the most well-established cellular targets of leptin, LepR is also expressed by non-neuronal cells throughout the body. Importantly, most immune cells, including hematopoietic bone-marrow precursors, monocytes/macrophages, and lymphocytes, express LepR, suggesting that leptin directly modulate immune responses and inflammation (La Cava and Matarese, 2004; Procaccini et al., 2012, 2017). Adipose tissue and lymphoid organs are often anatomically associated, and the contiguity between adipocytes and lymphoid cells supports the functional interactions (Matarese et al., 2002). For example, leptin affects thymic function and growth/survival of bone-marrow CD34^+^ precursors and CD4^+^ T cells (Lord et al., 1998; Howard et al., 1999; Martín-Romero and Sánchez-Margalet, 2001; Papathanassoglou et al., 2006; Cohen et al., 2017), and modulates both innate and adaptive immunity through diverse mechanisms (Maurya et al., 2018), such as enhancement of neutrophil oxidative burst (Mancuso et al., 2002; Caldefie-Chezet et al., 2003), phagocytosis by monocytes/macrophages (Faggioni et al., 1999; Fantuzzi and Faggioni, 2000; Sánchez-Margalet et al., 2003), and cytotoxic activity of natural killer (NK) cells (Tian et al., 2002), as well as mobilization of macrophages, lymphocytes (Abella et al., 2017), and neutrophils (Souza-Almeida et al., 2018).

Leptin deficiency and resistance are associated with increased susceptibility to infectious diseases, including bacterial (e.g., *Mycobacterium tuberculosis* and *Streptococcus pneumonia*), viral [e.g., coronaviruses, influenza A virus, and human immunodeficiency virus (HIV)], and parasitic (e.g., *Trypanosoma brucei*, *Trypanosoma cruzi*, and *Entamoeba histolytica*) infections (Amole et al., 1985; Sánchez-Pozo et al., 2003; Wieland et al., 2005; Hsu et al., 2007; Nagajyothi et al., 2010; Tschöp et al., 2010; Vedantama and Viswanathan, 2012; Zhang et al., 2013; Radigan et al., 2014; Alti et al., 2018; Guglielmi et al., 2021), while infection with certain pathogens, such as *Plasmodium* spp. and *Toxoplasma gondii*, can cause dysregulated leptin secretion, independently of adiposity (Pulido-Mendez et al., 2002; Baltaci and Mogulkoc, 2012). The significant association between obesity-induced inflammation (meta-inflammation) and the severity of infectious disease has been highlighted by COVID-19 pandemic (Huizinga et al., 2020; Rebello et al., 2020). It is notable that *Streptococcus pneumoniae* is a major cause of meningitis, which potentially leads to persistent cognitive disability (Yau et al., 2018), and *M. tuberculosis* can disseminate into the brain and induce CNS tuberculosis, a cascade of inflammatory responses that can potentially cause brain damage (Leonard, 2017; Davis et al., 2019). HIV-positive individuals frequently suffer neurocognitive disorders (HIV-associated neurocognitive disorders, HAND), and influenza A virus (H1N1) have been associated with neurological manifestation in both young and adult patients (Cárdenas et al., 2014; Wilking et al., 2014), with some developing permanent sequelae (Cárdenas et al., 2014), suggesting that defective leptin signaling could affect brain functions through increased infection susceptibility. While the mechanistic link between leptin resistance and infection susceptibility is complex and multifactorial (Maurya et al., 2018; Rebello et al., 2020), leptin’s actions through immune cells in the periphery and CNS, and dysregulation thereof, likely play a significant role.

### The Roles for Leptin and Insulin in Cellular Immunometabolism

Cellular energy metabolism is mainly driven by glycolysis, tricarboxylic acid (TCA) cycle, fatty acid oxidation, and oxidative phosphorylation (OXPHOS). Glutaminolysis, the conversion of glutamine to glutamate, is activated to fuel TCA cycle when glucose availability is limited. Glutaminolysis also plays a crucial role in the brain, where glutamate functions as a major neurotransmitter (Wang et al., 2017). In addition, in the absence of glucose, such as insulin-induced hypoglycemia, microglia utilize glutamine as an alternative fuel to support their immunological functions (Bernier et al., 2020). Upon stimulation by pathogen-derived molecules and endogenous ligands, immune cells undergo metabolic reprogramming into alternative modes of energy metabolism (O’Neill et al., 2016; Gaber et al., 2017; Hotamisligil, 2017; Próchnicki and Latz, 2017), which can be largely classified into the pro-inflammatory phenotypes dominated by glycolysis (similar to the Warburg effect in cancer cells), and anti-inflammatory phenotypes characterized by TCA cycle, fatty acid oxidation, and OXPHOS (O’Neill et al., 2016). Such bioenergetic shifts determine the properties of various immune cell populations, including macrophages (Van den Bossche et al., 2017), neutrophils (Curi et al., 2020), and T cells (O’Neill et al., 2016; Spadaro et al., 2017; Balyan et al., 2020), where insulin/IGFs and leptin come into play. For example, insulin regulates T cell’s metabolic reprogramming, thereby shaping adaptive immunity (Tsai et al., 2018). InsR-deficient T cells showed compromised responses to antigens *in vitro*, and T cell-specific InsR knockout in mice led to reduced antigen-specific immunity to influenza virus infection *in vivo* (Tsai et al., 2018), suggesting that InsR signaling reinforces metabolic reprogramming required for T cell activation. Such immunometabolic changes of T cells are likely mediated by mTOR, a key regulator of cellular homeostasis including protein synthesis and autophagy (Rao et al., 2010; Chi, 2012; Martin et al., 2021). mTOR also mediates insulin-induced alteration of metabolic rates and immune responses in myeloid cells (Ratter et al., 2021), and mTOR mediates age-associated microglial priming and neurodegeneration (Keane et al., 2021), suggesting that insulin-induced activation of mTOR pathway plays a role in immunometabolic imbalance in the CNS. IGF-1R signaling is essential for the anti-inflammatory polarization of macrophages upon metabolic stress as well as helminth infection (Labandeira-Garcia et al., 2017; Spadaro et al., 2017), and IGF-2 instructs macrophage precursor cells to become anti-inflammatory through metabolic pre-programming toward OXPHOS (Du et al., 2019). Leptin also induces immunometabolic changes in immune cells, including macrophages and T cells (Cohen et al., 2017; Boutens et al., 2018; Monteiro et al., 2019). Activation of leptin signaling pathways (JAK/STAT and IRS/PI3K) leads to intracellular metabolic changes, such as increased glucose uptake and glycolytic activity and reduced OXPHOS, associated with pro-inflammatory phenotype of macrophages and T cells (Cohen et al., 2017; Boutens et al., 2018; Monteiro et al., 2019). It is conceivable that immunometabolic imbalance due to dysregulated insulin/IGF and leptin signaling pathways underlie immunodeficiency and infection susceptibility in nutritional imbalance, including malnutrition and obesity.

### Brain-Resident Immune Cells and Immunometabolism

#### Microglia and Astrocytes

**FIGURE 2:**
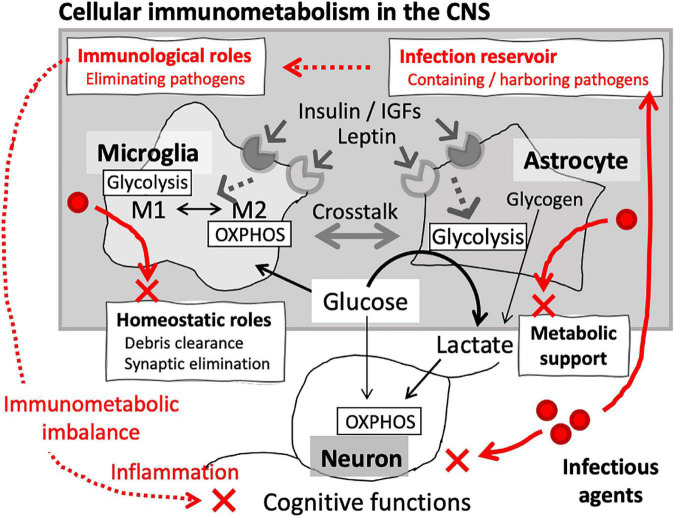
Cellular immunometabolism in the brain. Glia are the main cellular components of immunometabolism in the CNS. Microglial immunological phenotypes are closely associated with their metabolic signature: glycolysis-dominant proinflammatory (M1) and OXPHOS-dominant anti-inflammatory (M2) phenotypes. In addition to the role as resident innate immune cells, microglia also play homeostatic roles, such as eliminating unwanted synapses and debris. Astrocytes are the major metabolic component of CNS, providing bioenergetic support for the neuronal networks. Microglia and astrocytes express InsR and LepR, through which their metabolic signatures and immunological phenotypes are regulated. Crosstalk between microglia and astrocytes constitutes cellular immunometabolism in the CNS. Infection can disrupt the immunometabolic homeostasis, which may lead to neuronal damages and cognitive impairment.

The brain is the most energy-demanding organ, consuming glucose at a disproportionately high rate compared to the rest of the body. Glucose metabolism in the brain is founded on intricate interactions between the cellular components, i.e., neurons and glia. Neurons are highly aerobic and heavily dependent on OXPHOS, while astrocytes and oligodendrocytes, the major neuroglial populations, are predominantly glycolytic. These cell-type specific metabolic profiles support the overall power system in the CNS: lactate generation via glycolysis in neuroglia fueling OXPHOS in neurons (Fünfschilling et al., 2012; Jha and Morrison, 2018; Rosko et al., 2019) (Figure 2). To protect such elaborate systems, the brain harbors resident innate immune cells, namely resident macrophage populations: microglia, perivascular macrophages, meningeal macrophages, and choroid plexus macrophages. The former three, including microglia, have embryonic origins and are maintained through local self-renewal while choroid plexus macrophages are replenished via constant supply of bone marrow-derived monocytes (Prinz et al., 2017). These brain-resident macrophage populations play significant roles in the brain physiology, as well as in defense against infection (Ransohoff and Brown, 2012; Heneka et al., 2015; Herz et al., 2017; Kierdorf and Prinz, 2017).

While aberrant activation of resident macrophages contributes to CNS pathology via inflammation (De Strooper and Karran, 2016; Herz et al., 2017), evidence is growing that brain-resident macrophages play homeostatic roles in the brain. Residing in the brain parenchyma, microglia play pivotal roles in the development and maintenance of neuronal networks. The most notable roles of microglia include complement-dependent synaptic pruning during CNS development (Schafer et al., 2012) and synaptic reorganization throughout life (Herz et al., 2017). In addition, microglia-derived neurotropic factors, such as brain-derived neurotrophic factor (BDNF), promote learning-dependent synaptic formation (Parkhurst et al., 2013) and protect neurons from brain injuries (Madinier et al., 2009). Furthermore, CNS macrophages are the major component of glymphatic system, which plays a crucial role in the clearance of waste products and toxic materials, including Aβ (Gordleeva et al., 2020). In the context of AD pathogenesis, microglial activation is a double-edged sword; it can facilitate the clearance of Aβ and tau, while potentially inducing neuroinflammation leading to neuronal damages associated with AD (Lue et al., 2010; Leyns and Holtzman, 2017; Shippy and Ulland, 2020).

Meanwhile, growing evidence suggests that astrocytes are part of the innate immunity in the brain (Sofroniew, 2020). Responding to the changing microenvironment and diverse stimuli (e.g., microbial infections, exposure to environmental toxins, tumor formation, and neurodegenerative diseases), astrocytes undergo morphological and functional changes and influence the outcome of a number of brain disorders (Drögemüller et al., 2008; Soung and Klein, 2018; Katsouri et al., 2020; Sofroniew, 2020). In addition, crosstalk between astrocytes and microglia in the hippocampus is responsible for sensitivity to insults (Lana et al., 2020), pointing to the importance of communication between astroglia and brain-resident macrophages in cognitive dysfunctions (Liddelow et al., 2017; Linnerbauer et al., 2020). Furthermore, astrocytes and microglia participate in Aβ clearance and protection of synaptic connectivity in AD models, suggesting highly context-dependent immunological roles of these glial cell populations against AD development (Davis et al., 2021).

#### Glial Cells and Central Nervous System Immunometabolism

Microglia and astrocytes, also called macroglia, are major resident non-neuronal cells in the brain. As described above, evidence suggests that the interplay between neurons and these glial population plays pivotal roles in the brain, including the hippocampus, in physiological and pathological conditions (Lana et al., 2021). While neuronal cells express InsR/IGF-1R at high levels and many studies have focused on the roles of neuronal InsR/IGF-1R (Frölich et al., 1998), microglia and astrocytes also express InsR and IGF-1R (Shaughness et al., 2020). Insulin and IGFs support brain functions through regulating astroglial glucose metabolism (Sonnewald et al., 1996; Fernandez et al., 2018). InsR signaling modulates astroglial glucose uptake and bioenergetics (Heni et al., 2011), and specific ablation of astroglial InsR led to altered mood and cognition in mice (González-García et al., 2021). In addition, insulin can modulate inflammatory responses of astrocytes (Spielman et al., 2015), suggesting the immunometabolic regulation of astrocytes by insulin. IGF-1R can also modulate astroglial metabolic and immunological signatures (Hernandez-Garzón et al., 2016), and astroglial IGF-1R signaling mediates synaptic plasticity of cortical inhibitory neurons (Noriega-Prieto et al., 2021). These data suggest that impairment in InsR/IGF-1R signaling pathways may disrupt the metabolic network between glia and neurons, as well as immunological roles of astrocytes, eventually leading CNS dysfunctions. Microglial InsR/IGF-1R also mediate the effects of insulin. Low-dose insulin exerted pro-inflammatory effects on microglial cells *in vitro* (Spielman et al., 2015), while it was anti-inflammatory at a higher concentration (Brabazon et al., 2018), suggesting the variable roles of microglial InsR/IGF-1R signaling. In addition, microglia are the major source of IGF-1 in the brain, and microglial IGF-1 was found to be increased in AD model mice compared to wild-type (Myhre et al., 2019). These data suggest that insulin and IGFs play crucial roles in immunometabolism via the phenotypic regulation of glial cells, potentially mediating the link between bioenergetics and immunity in the brain.

Microglia (Tang et al., 2007; André et al., 2017; Fujita and Yamashita, 2019), and astrocytes (Naranjo et al., 2020; Pratap and Holsinger, 2020) express LepR. Leptin can enhance microglial pro-inflammatory responses, including IL-6 production through a mechanism involving insulin receptor substrate-1 (IRS-1), PI3K, and Akt (Tang et al., 2007; André et al., 2017; Fujita and Yamashita, 2019), IL-1β release via a caspase 1-independent mechanism (Pinteaux et al., 2007), and lipopolysaccharide (LPS)-induced pro-inflammatory responses (Lafrance et al., 2010). On the other hand, in a spinal cord injury model, leptin reduced microglial inflammatory responses, while inducing neuroprotective phenotypes, Fernández-Martos et al. (2012). In myeloid cell-specific LepR deficient mice, hypothalamic microglia exhibited less ramified morphology and impaired phagocytic capacity (Gao et al., 2018), suggesting that leptin directly regulates homeostatic microglial phenotypes (Davis et al., 2017). Considering multifaceted physiological roles of microglia and potential harm (i.e., neuroinflammation) caused by their aberrant activation (Colonna and Butovsky, 2017; Li and Barres, 2018), it is conceivable that impaired leptin signaling in microglia could lead to significant homeostatic imbalance in the CNS. On the other hand, specific LepR depletion in astrocytes (GFAP-LepR–/–) resulted in impaired neurotransmission in the hippocampus, suggesting that leptin regulates hippocampal plasticity via astroglial LepR, possibly by regulating the glucose and glutamate up-take capacity (Naranjo et al., 2020).

#### Other Central Nervous System-Resident Immune Cells

In addition to resident macrophages, the CNS harbors multiple leukocyte populations, including dendritic cells (DCs) and T lymphocytes. As a professional antigen-presenting cells, DCs play an essential role in the regulation of adaptive immunity. Although initially believed to be absent in the healthy brain parenchyma, only appearing in response to aging, injury, and infections (Fischer and Reichmann, 2001), studies have shown the presence of DCs within the healthy steady-state brain (Bulloch et al., 2008; Prodinger et al., 2011). Upon activation, brain DCs can migrate out of the brain and induce T-cell homing into the CNS where antigen-specific immune responses may take place (Karman et al., 2004). Correlation between aging and the accumulation of DCs in the CNS (Kaunzner et al., 2012) suggests potential involvement of brain DCs in immunological changes and inflammation associated with aging. In response to immunological stimuli, such as viral and parasitic infections, T cells infiltrate into the brain, where these cells play beneficial roles by eliminating pathogens. In addition, studies have shown that long-lived memory T cells are established after CNS infections (Wakim et al., 2012; Landrith et al., 2017; Mockus et al., 2018), and these tissue-resident memory CD8 cells can provide frontline defense against re-infection (Netherby-Winslow et al., 2021). However, persistence of these cells can also cause neuronal damages (Ai and Klein, 2020; Ghazanfari et al., 2021).

Although it is unknown how these CNS resident immune cells respond to and regulated by leptin and insulin, InsR is expressed on the surface of activated T cells (Helderman et al., 1978). Acting through InsR, insulin enhances and maintains T cell functions after immunological challenges (Helderman, 1984) and potentially induces anti-inflammatory polarization in the periphery (Viardot et al., 2007). These data suggest that insulin may play an immunomodulatory role via actions on T cell populations in the brain.

#### Glia as the Target and Reservoir of Infectious Agents

**FIGURE 3:**
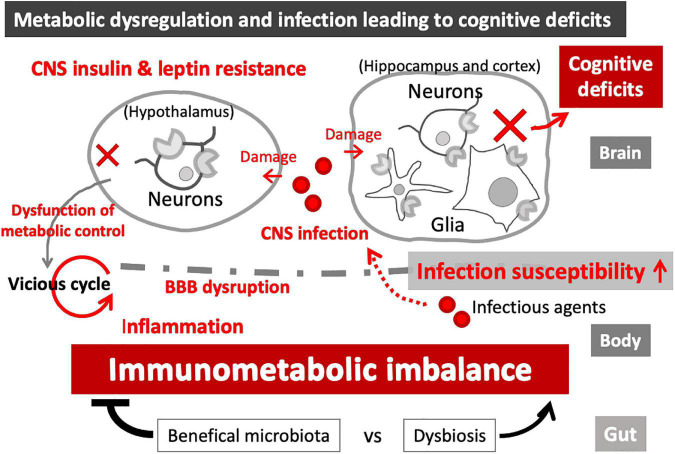
Immunometabolic imbalance in the CNS. Leptin and insulin resistance, associated with metabolic disease, can cause immune dysfunctions and increased infection susceptibility. Metabolic imbalance also causes BBB disruption, leading to neuroinflammation and loss of CNS homeostasis, eventually resulting in functional damages to the affected brain regions. While hypothalamic damages can magnify immunometabolic imbalance in the periphery (vicious cycle), hippocampal and cortical damages can cause cognitive impairment. CNS infection-induced brain damages can further enhance the immunometabolic imbalance and cognitive deficits, as part of the vicious cycle.

Growing evidence supports the infectious etiology of dementia, and a number of causative agents have been proposed, including viruses (e.g., Herpes simplex virus type 1 (HSV-1), cytomegalovirus (CMV)] and bacteria (e.g., *Chlamydophila pneumoniae*, spirochetes, *Helicobacter pylori*, *Porphyromonas gingivalis*) (Harris and Harris, 2015; Sochocka et al., 2017; Nazareth et al., 2021). Suggested mechanisms include persistent inflammation caused by chronic infection, leading to a vicious cycle of neuroinflammation and neurodegeneration, as well as BBB disruption, which may further enhance the entry of infectious agents and proinflammatory mediators from the periphery (Sochocka et al., 2017) (Figure 3).

Some infectious agents can enter and persistently inhabit the brain, directly causing chronic activation of resident immune cells (Sochocka et al., 2017). For example, *Chlamydia pneumoniae*, obligate intracellular bacteria, are frequently found in microglia and astrocytes in the brain of AD patients (Balin et al., 1998; Nazareth et al., 2021). Microglia and astrocytes are also the target of some viruses, such as CMV (Cheeran et al., 2001) and HIV (Avalos et al., 2017; Valdebenito et al., 2021). In addition, *T. gondii*, a neurotropic protozoan parasite causing a lifelong CNS infection, has been implicated in various neurological disorders, including dementia (Nayeri Chegeni et al., 2019). *T. gondii is* an obligate intracellular parasite, potentially infecting all nucleated cell types. Chronic *Toxoplasma* infection is linked to microglial activation and persistent neuroinflammation (Li et al., 2019), and CNS energy metabolism is significantly skewed toward glycolysis upon infection (Hargrave et al., 2019), suggesting that *T. gondii* interferes with host immunometabolism in the brain. Indeed, it has been shown that *Toxoplasma* infection may lead to reduced IGF-1 signaling in the brain (El Saftawy et al., 2020). On the other hand, *In vitro*, high glucose and up-regulation of glycolysis in the host cell had a significant impact on the life-stage conversion of *T. gondii* (i.e., from fast-growing tachyzoite stage into dormant bradyzoite stage) (Weilhammer et al., 2012), indicating that host cell metabolism could modulate the virulence of intracellular pathogens. Intriguingly, *T. gondii* infection can enhance Aβ clearance by increasing phagocytic activity of recruited monocytes (Möhle et al., 2016), suggesting that the ultimate outcome may depend on the extent and the aggressiveness of the infection. Of note, SARS-CoV-2 is frequently found in astroglia and microglia, rather than neurons, in the autopsy brain, indicating potential involvement of glial activation in the neurological manifestation of COVID-19 (Solomon, 2021). These data suggest that infectious agents could produce immunometabolic imbalance in the CNS via affecting microglial and astroglial phenotypes (Figure 2), which may lead to chronic inflammation and an increased risk of neurodegenerative disorders (Stephenson et al., 2018).

## Insulin and Leptin Mediating the Link Between Infection and Neurodegenerative Disorders

### Infectious Etiology of Dementia

Recently, it has been shown that Aβ, a hallmark of AD, is an antimicrobial peptide, part of innate immunity to protect the host from various infectious agents (Soscia et al., 2010; Kumar et al., 2016; Gosztyla et al., 2018), suggesting the necessity to revisit the role of amyloid plaque formation in AD pathogenesis. Above mentioned neurotropic viruses, such as HSV-1, HSV-2 and CMV, certain bacterial species, including spirochetes and *P. gingivalis*, fungi (e.g., *Candida albicans*), and neurotropic parasite (e.g., *T. gondii*), may cause disruption of brain functions via chronic inflammation and immune dysregulation in the brain (Sochocka et al., 2017; Shinjyo et al., 2021). The antimicrobial hypothesis for AD proposes that Aβ generation and amyloid plaque formation are not the major culprit in AD pathogenesis, but rather a defense against such infectious agents (Moir et al., 2018; Iqbal et al., 2020; Fulop et al., 2021).

While metabolic disorders and infection may independently increase the risk of dementia, obesity and metabolic disorders may indirectly increase the risk of infection-induced neurological disorders, by enhanced susceptibility to infection and infection-induced complications via immune dysregulation (Shah and Hux, 2003; Muller et al., 2005; Knapp, 2013; Tamara and Tahapary, 2020). Conversely, obesity can occur as a result of infection (Pasarica and Dhurandhar, 2007; Dhurandhar, 2011), and infection-induced chronic low-grade inflammation may lead to insulin resistance (Fernández-Real et al., 2006), suggesting bi-directional interactions between infection and metabolic disorders. Furthermore, several pathogens, such as HSV, CMV, *H. pylori*, and *P. gingivalis*, are implicated in both metabolic disorders and AD (Jeon et al., 2012; Harris and Harris, 2015; Fleck-Derderian et al., 2017; Beydoun et al., 2018; Lövheim et al., 2018; Mei et al., 2020; Baradaran et al., 2021; Costa et al., 2021; Salem et al., 2021), supporting the potential mechanistic link between infection, MetS, and dementia etiology.

### Metabolic Imbalance Causing Central Nervous System Infection Susceptibility

Studies showing possible interactions between metabolic imbalance and infection in dementia etiology, particularly focusing on data highlighting the roles for insulin and leptin, are listed in Table 1.

**TABLE 1:** The roles of insulin and leptin in central nervous system (CNS) infection.

Pathogen/host	Study design and outcome measures	Findings	Proposed direction of causality and/or mechanism	References
**Borna disease virus (BDV)**				
BDV/mouse	Animal model: virus-induced obesity model using intracerebral injection of BDV-1 in rats. Outcome measures: neuropathology including inflammatory infiltrates and neurodegeneration in the hypothalamus, hippocampal shrinkage, and **leptin** resistance	BDV infection-induced obesity was associated with: 1. hypothalamic inflammation. 2. Hippocampal involution and microglial activation in the neocortex. 3. Preferential infection of glutamatergic sites, while sparing GABAergic areas, causing (anabolic appetite-stimulating) GABAergic predominance and fat accumulation 4. **Leptin** resistance in the brain.	CNS infection → Hypothalamic inflammation, neurotransmitter imbalance (GABA predominance), obesity, and **leptin** resistance.	Gosztonyi et al., 2020
BDV/rat	Animal model: BDV infection models using two different strains: *BDV-ob* (obesity-inducing) and *BDV-bi* (no obesity-inducing effect). Outcome measures: Mononuclear infiltrates into the brain, astrogliosis, and neuronal death.	1. *BDV-ob* infection and mononuclear infiltrates were restricted to certain brain areas including hypothalamus, hippocampus, and amygdala. Particularly severe infiltration in the median eminence of hypothalamus. (*BDV-ob* infection was observed evenly throughout the brain.) 2. Mononuclear infiltrates, astrogliosis, and neuronal death in the hippocampus of *BDV-ob* infected brain.	CNS infection →Hypothalamic inflammation and disruption of neuroendocrine system →Obesity	Herden et al., 2000
**Canine distemper virus (CDV)**				
CDV/mouse	Metabolic disturbance: obesity induced by intracerebral CDV infection. Outcome measures: Plasma **insulin** and lipid composition.	**Hyperinsulinemia** and triglyceride accumulation in CDV-induced obesity mice	CDV infection → **Hyperinsulinemia** and obesity	Bernard et al., 1988
CDV/mouse	Animal model: obesity induced by intracerebral infection with CDV. Outcome measures: **Leptin** and **LepR** expression.	Functional **LepR** was specifically downregulated in the hypothalamus of obese mice.	CNS infection →**Leptin** resistance in the hypothalamus. → Obesity.	Bernard et al., 1999
**Human immunodeficiency virus (HIV)**				
HIV/human	Subjects: patients with HIV-1 infection (n203, Cohort study). Outcome measures: association between HIV-associated dementia (HAND) and **diabetes.**	HAND was significantly associated with **diabetes** (odds ratio 5.43, 1.66–17.70), which was not fully explained by age or coexisting vascular risk factors.	**Diabetes** ↔ HAND	Valcour et al., 2005
HIV/mouse	Animal model: HIV model in mice using EcoHIV, with vs. without intranasal **insulin** treatment (daily for 9 days). Outcome measures: cognitive functions, hippocampal neuronal integrity, and the expression of genes associated with brain functions.	1. Infected mice exhibited cognitive impairment. 2. Intranasal **insulin** restored cognitive functions, hippocampal dendritic integrity, and gene expressions. 3. The beneficial effect of intranasal **insulin** was independent of HIV burden in the brain.	Infection →Cognitive impairment. Central **insulin** treatment → Restoration of brain functions	Kim et al., 2019
HIV/human FIV/cat	1. Human *ex vivo* model: (1) brain autopsy of patients with HIV/AIDS. (2) HIV-1 infection in primary human neurons and microglia, treated with **insulin**. Outcome measures: neuroinflammation and neuronal death. 2. Animal *in vivo* HAND model: feline retrovirus (FIV) intracranial infection in cats. **Insulin** intranasal treatment for 6 weeks compared to PBS treatment. Outcome measures: morphological changes in the brain, neuroinflammation, neuronal survival, neurobehavioral performance.	1-1. Increased neuroinflammatory gene expression in the brain of HIV/AIDS. 1-2. **Insulin** treatment suppressed HIV-1 growth and reduced infection-induced *CSCL10* and *IL-6* expression in HIV-infected microglia. 1-3. **Insulin** treatment prevented HIV-1 infection-induced neuronal death. 2-1. **Insulin** treatment enhanced the preservation of cortical neurons, and improved neurobehavioral performance in FIV-infected cats.	Infection → Cognitive impairment. Central **insulin** treatment → Restoration of brain functions.	Mamik et al., 2016
***Porphyromonas gingivalis***				
*P. gingivalis*/mouse	Animal model: ***db/db*** mouse infected with *P. gingivalis* (W83, p.o.). Outcome measures: neuroinflammation in the hippocampus; mRNA levels for genes associated with **insulin** signaling.	1. Infection induced reactive microglia and astrocytes. 2. Infection enhanced the expression of insulin signaling pathway genes (e.g., **InsR**, **Igf1**, **Irs**, and Gsk3β). 3. Pro-inflammatory genes were also up-regulated.	Infection → Disruption of **insulin** signaling pathway and inflammation in the brain.	Bahar et al., 2021
***Toxoplasma gondii***				
*T. gondii*/rat	Animal model: *T. gondii* infection model in rats. Outcome measures: plasma **leptin** levels	Plasma **leptin** levels increased in chronic *T. gondii* infection.	CNS infection → Increase in plasma **leptin** (metabolic imbalance)	Baltaci and Mogulkoc, 2012
**West Nile virus (WNV)**				
WNV/mouse	Animal model: ***db/db*** mouse infected with WNV. Outcome measures: leukocyte infiltration and neuroinflammation/neuronal damage.	Infection-induced leukocyte infiltration into the brain was reduced, while neuroinflammation/neuronal death was enhanced, in ***db/db*** mice.	**LepR** dysfunction → Increased CNS infection susceptibility	Kumar et al., 2014

*Relevant keywords, such as insulin and leptin, are highlighted in bold.*

#### West Nile Virus

West Nile virus (WNV) is a single-stranded RNA virus, genetically related to the Japanese encephalitis virus (JEV). Transmitted by mosquitoes, WNV potentially causes life-threatening encephalitis or meningitis, especially in the elderly (Alli et al., 2021). In addition, it can cause persistent cognitive impairments (Murray et al., 2014; Vittor et al., 2020). While CNS infiltration of lymphocytes, including CD8^+^ T cells, is essential in eliminating viruses, persistent inflammation can cause synaptic loss and neuronal death, impairing cognitive ability (Garber et al., 2019; Vittor et al., 2020). Of note, diabetes is a frequent comorbidity of severe WNV diseases and considered a risk factor for developing WNV encephalitis (Badawi et al., 2018). In *db/db* obesity model, WNV infection-induced leukocyte infiltration into the brain was significantly lower, suggesting that obesity compromises protection against viral infection in the brain (Kumar et al., 2014). In particular, infiltration of CD8^+^ T cells was significantly reduced in obese mice, which was associated with higher viral load and enhanced inflammatory responses in the brain (Kumar et al., 2014), suggesting a role of leptin signaling in protecting the brain via leukocyte recruitment (Rummel et al., 2010).

#### Porphyromonas gingivalis

*Porphyromonas gingivalis* is a periodontal disease-causing Gram-negative bacteria found in the oral cavity. Evidence suggests a strong association between *P. gingivalis* infection and sporadic AD (Kanagasingam et al., 2020), and its presence has been identified in the brain of AD patients (Dominy et al., 2019). *P. gingivalis* infection can trigger inflammation both in the periphery and CNS in affected individuals, leading to cognitive decline (Dominy et al., 2019; Kanagasingam et al., 2020). In mice, oral *P. gingivalis* infection led to brain colonization and AD-like pathogenesis, including complement activation and Aβ_1__–__42_ formation (Poole et al., 2015; Dominy et al., 2019), suggesting potential mechanistic links between periodontal disease and AD. A systematic review of pre-clinical studies also found that *P. gingivalis* infection induced inflammatory responses and tissue degeneration in the brain, which were associated with cognitive impairment (Costa et al., 2021).

Periodontitis is also associated with obesity (Suvan et al., 2011; Keller et al., 2015; Nascimento et al., 2015; Khan et al., 2018). High fat diet (HFD) significantly enhanced systemic inflammation induced by periodontal pathogens in rodents (Virto et al., 2018). *P. gingivalis* infection up-regulated the expression of genes associated with insulin/IGF-1 signaling and induced inflammatory responses in the brain of *db*/*db* mice (Virto et al., 2018), suggesting that metabolic imbalance due to dysregulated insulin and leptin signaling pathways may exacerbate the outcome of periodontitis and associated inflammation in the brain.

#### Human Immunodeficiency Virus

Despite the effectiveness of antiretroviral therapy in saving the lives of many from acquired immunodeficiency syndrome (AIDS), HIV-positive individuals frequently suffer neurocognitive disorders (HAND) (Antinori et al., 2007; Foley et al., 2011). While inflammation and brain atrophy due to persistent viral presence in the CNS may partly provide explanations (Pemberton and Brew, 2001; Woods et al., 2010; Ances et al., 2012; Brew and Barnes, 2019), exact mechanisms underlying HAND remain unclear.

Metabolic syndromes, including diabetes, is prevalent in HIV-infected individuals (Calza et al., 2011; Paik and Kotler, 2011), and insulin resistance was associated with lower cognitive scores in a HIV-1 cohort (Valcour et al., 2005, 2006), suggesting that HIV infection may increase the risk of cognitive impairment via metabolic dysregulation. In fact, in a murine model of HAND, intranasal insulin administration restored hippocampal dendritic integrity and cognitive functions, independently of HIV burden in the brain (Kim et al., 2019). In primary human neurons and microglia *in vitro*, insulin suppressed infection-induced inflammatory responses and HIV-1 growth in microglia, and prevented infection-induced neuronal death (Mamik et al., 2016). In addition, in a feline HIV model *in vivo*, intranasal insulin enhanced the preservation of cortical neurons and improved cognitive performance (Mamik et al., 2016), suggesting that impaired insulin signaling in the CNS may underlie cognitive impairment in HIV positive individuals.

### Central Nervous System Infections Causing Metabolic Imbalance – Borna Disease Virus and Canine Distemper Virus

Borna disease virus (BDV) is a neurotropic RNA virus infecting a broad host spectrum including humans. Borna disease was originally observed as infectious diseases in domestic animals in the nineteenth century. The confirmation of human infection has begun since the 1980s, mainly in neuropsychiatric patients (Hatalski et al., 1997; Schwemmle, 2001; Taieb et al., 2001). BDV causes persistent infection in the brain and neurobehavioral deficits associated with neuroinflammation. In an experimental infection model in rodents, it takes a biphasic course characterized by hyperactivity associated with inflammatory lesions in the brain during the first acute stage, followed by the development of varying symptoms, including obesity, depending on viral strains and affected brain regions (Narayan et al., 1983; Herden et al., 2000). Intracerebral infection of rats with a BDV variant induced obesity without neurological signs, which is correlated with severe mononuclear cell infiltration into the hypothalamus, suggesting that infection-induced neuroendocrine dysregulations caused the development of obesity (Herden et al., 2000). It was also suggested that BDV infection-induced neuroinflammation and neurotransmitter imbalance underlie the dysfunction of hypothalamus and leptin resistance, leading to obesity (Gosztonyi et al., 2020).

Canine distemper virus (CDV) is an RNA virus closely related to measles virus, infecting a wide range of host species (Martinez-Gutierrez and Ruiz-Saenz, 2016). CDV causes canine distemper, a severe systemic disease in dogs, presenting a variety of symptoms including neurologic disorders (Martella et al., 2008). In a virus CDV infection-induced obesity model in mice using intracerebral infection, hyperinsulinemia and alteration in leptin signaling were observed (Bernard et al., 1988, 1999). In this model, infection caused hyperinsulinemia and obesity, while CDV showed tropism for the hypothalamus. Obesity developed in up to 30% of the surviving mice (Bernard et al., 1988). In addition, functional LepR was specifically down-regulated in the hypothalamus of infected obese mice (Bernard et al., 1999), suggesting that CDV infection in the brain induced leptin resistance in the hypothalamus, which led to obesity, thereby increasing the risk of MetS-associated cognitive impairment (Figure 3).

### Peripheral Infection and Cognitive Impairment – Potential Involvement of Insulin and Glycemic Control

Peripheral infection-induced metabolic disturbance can lead to CNS dysfunction (Table 2). Sepsis-associated encephalopathy (SAE) is a brain disease secondary to peripheral infection without overt CNS infection, occurring up to 50–70% of sepsis cases (Chen Q. et al., 2014). After initial response to eliminate pathogens, systemic inflammation and increased BBB permeability occur, causing severe encephalopathy. While SAE is partly reversible, it can lead to persistent neurocognitive deficits, increasing the risk of dementia later in life (Widmann and Heneka, 2014; Seidel et al., 2020). Disrupted glycemic control is frequently encountered in sepsis patients (Hirasawa et al., 2009), and it has been suggested that higher infection susceptibility in patients with hyperglycemia is associated with disease severity (Koh et al., 2012). Experimental sepsis in rodents induced cognitive deficits accompanied by hyperglycemia (Huang et al., 2020). BBB disruption, microglial activation, oxidative damage and inflammation in the hippocampus, cortex and cerebrum occurred in those animal models (Michels et al., 2015; Sonneville et al., 2015; Huang et al., 2020), suggesting glial activation and neuroinflammation underlie SAE-induced cognitive impairment. In addition, the experimental sepsis induced more severe brain damages, including microglial activation and neuronal death, in hyperglycemic mice compared to insulin-treated mice (Sonneville et al., 2015), suggesting that poor glycemic control renders CNS more vulnerable to neuroinflammation, and insulin may protect the brain from sepsis-induced neuroinflammation and neuronal damages (Hache et al., 2015).

**TABLE 2:** The roles of insulin in peripheral infection-induced CNS damage.

Pathogen or disease/host	Study design and outcome measures	Findings	Proposed direction of causality and/or mechanism	References
Sepsis/rat	Animal model: surgically induced polymicrobial sepsis model in rats. Glucose treatment, **insulin** treatment compared to control. Outcome measures: Blood glucose, behavioral deficits, brain activity (EEG), BBB permeability, glial activation and inflammation in the cerebrum.	1. Sepsis induced hyperglycemia. 2. Glucose treatment led to a decline in survival rate, reduced brain activity, increased BBB permeability, and enhanced microglial and astroglial activation and inflammatory responses in the cerebrum. 3. Glycemic control (**insulin** treatment) inhibited inflammatory responses and restored BBB integrity and brain activity to near normal.	Peripheral infection →Hyperglycemia. →Glial activation and neuroinflammation. → Cognitive dysfunction. Note: **Insulin** help restore brain functions by preventing BBB disruption and neuroinflammation.	Huang et al., 2020
Sepsis/mouse	Animal model: surgically induced polymicrobial sepsis model in mice. Mice with moderate hyperglycemia were compared to control (normoglycemia). Outcome measures: neuronal damages, glial activation, and cell death in the hippocampus and frontal cortex.	(A) In hyperglycemic mice (compared to normoglycemia mice), sepsis induced: 1. Higher neuronal damage in frontal cortex. 2. Microglial activation in frontal cortex and hippocampus. 3. More apoptotic cells in frontal cortex. (B) **Insulin** prevented the above damages	Hyperglycemia →Enhanced infection (sepsis)-induced brain damage. Note: **Insulin** prevents hyperglycemia-induced susceptibility to sepsis-induced brain damages	Sonneville et al., 2015
Sepsis/mouse	Animal model: surgically induced polymicrobial sepsis model in mice. Outcome measures: cognitive functions, hippocampal synaptic plasticity, and hippocampal insulin signaling in post-septic mice compared to control.	1. Post-septic mice exhibited cognitive impairment, which was accompanied by reduced synaptic plasticity and disrupted insulin signaling in the hippocampus. 2. Treatment with a GLP-1 receptor agonist (**insulinotropic**) or GSK3β inhibitor (**insulin** signaling downstream) rescued cognition.	Peripheral infection →Disruption of hippocampal **insulin** signaling. → Impaired synaptic plasticity and cognitive deficits.	Neves et al., 2018
Sepsis/rat	Animal model: sepsis model induced by LPS (i.p.) in rats. **Insulin** treatment: continuous intravenous infusion for 6h after LPS stimulation. Compared to control (saline). Outcome measures: Inflammatory cytokines and oxidative stress in the cortex, hippocampus, and hypothalamus.	In the brain regions (cortex, hippocampus, and hypothalamus): 1.**Insulin** alleviated sepsis-induced inflammatory response (IL-1β, IL-6, and TNF-α). 2. **Insulin** suppressed oxidative damage while restoring antioxidants (SOD and GSH).	Peripheral infection →Inflammation and oxidative stress in the brain. Note: **Insulin** treatment lowered sepsis-induced inflammation in the brain	Chen Q. et al., 2014
Systemic inflammation/mouse	Animal model: LPS (i.p.) challenge in chronic neurodegeneration model (ME7 prion infection) in mice. Treated with glucose and **insulin**. Outcome measures: blood glucose, cognitive performance	LPS challenge induced hypoglycemia and acute cognitive impairment in mice with brain disease (ME7 prion infection), which was mitigated by glucose and mimicked by **insulin**.	Peripheral infection →Metabolic imbalance in the CNS. →Cognitive impairment. Note: Brain disease (prion disease) makes the CNS more vulnerable to peripheral inflammation	Kealy et al., 2020

*Relevant keywords, such as insulin and leptin, are highlighted in bold.*

In addition, acute inflammation in the periphery (LPS challenge) induced metabolic changes in the CNS in rodents, and the metabolic imbalance in the brain was associated impaired cognition (Kealy et al., 2020). In addition, LPS-induced acute peripheral inflammation produced hypoglycemia in blood and CSF, and caused severe cognitive impairment selectively in those mice with brain disease (chronic neurodegeneration model induced by prion infection), which was mimicked by insulin and alleviated by glucose administration (Kealy et al., 2020). These data suggest that acute peripheral inflammation can cause neuroinflammation and cognitive deficits via disruption of glycemic control, whereas chronic neuroinflammation, including infection-induced neurodegenerative disorders, renders the brain more vulnerable to metabolic imbalance (Figure 3).

### Other Pathogens Affecting Metabolic Balance – Indirect Link to Cognition

‘Infectobesity’ is a concept that proposes the infectious etiology of obesity (Pasarica and Dhurandhar, 2007; van Ginneken et al., 2009). Considering the link between MetS and cognitive decline (Mejido et al., 2020), infectobesity can be an indirect cause of dementia. In addition to BDV and CDV, there are several viruses potentially causing obesity (Dhurandhar, 2001; Atkinson, 2007). Rous-associated virus 7 (RAV-7), a retrovirus causing avian leukosis, can induce stunting and obesity in chickens, which is associated with hyperlipidemia and increased insulin levels, as well as immune dysregulation (Carter and Smith, 1984). Adenovirus 36 (Ad-36) is a human adenovirus associated with obesity (Esposito et al., 2012). Ad-36 infection in adipocytes reduced leptin production while inducing proliferation, differentiation, and lipid accumulation in adipocytes (Vangipuram et al., 2007; van Ginneken et al., 2009), suggesting viral infection of fat cells can directly cause adipogenesis and obesity. Reduced leptin release caused by infection may also compromise CNS-mediated control of energy homeostasis, further enhancing the risk of MetS-associated cognitive impairment (Whitmer et al., 2005; Kivipelto et al., 2006; Mejido et al., 2020).

### Gut Microbiome

While the concept of microbial infection and pathogenicity has dominated the mainstream of microbiology, only a small fraction of microorganism are inherently pathogenic. In particular, the gut is inhabited by microbiota, a collection of microorganisms including bacteria, archaea, viruses, and fungi, consisting of at least 1,000 distinct species. Commensal bacterial and fungal species are involved in the regulation/dysregulation of energy homeostasis and immune responses, through extracting and metabolizing nutrients, regulating peripheral and central insulin sensitivity (Schertzer and Lam, 2021), and contributing to intestinal immune control via bidirectional communication with immune cells (Khan et al., 2021). Disruption of the symbiotic relationship between the host and microbiota leads to chronic inflammation and insulin resistance (Patterson et al., 2016). Obesity is associated with altered gut microbial composition in mice and humans, and the trait is transmissible as colonization of germ-free mice with microbiota from obese mice led to a significant increase in body fat compared to colonization with microbiota from lean mice (Turnbaugh et al., 2006), suggesting that gut microbiome is part of the host metabolic system actively regulating energy balance (Bäckhed et al., 2004). It has also been suggested that microbiome plays a crucial role in the communication between the gut and brain (microbiota-gut-brain axis), which is essential for the regulation of energy homeostasis (Romaní-Pérez et al., 2021; van Son et al., 2021) as well as the development and functions of the nervous system (Chen et al., 2021; Gwak and Chang, 2021). Consequently, altered gut microbiota (dysbiosis) has been implicated in a number of chronic inflammatory diseases, such as diabetes (Zawada et al., 2020; Rodriguez and Delzenne, 2021) and neurodegenerative disorders, including AD (Jiang et al., 2017; Chen et al., 2021; Leblhuber et al., 2021; Romanenko et al., 2021). Furthermore, these indigenous microbiota play a critical role in host defense against infection, through stimulating mucosal immune defenses (e.g., of antimicrobial peptides and IgA release) and limiting resource availability/niche opportunity for invading microbes (Libertucci and Young, 2019). Consequently, dysbiosis has been associated with infection susceptibility (Lazar et al., 2018; Libertucci and Young, 2019), which may further accelerate immunometabolic imbalance.

Probiotics are living microorganisms that provide health benefits by improving or restoring the composition of gut microbiota. It has been shown that probiotics can reduce leptin secretion and improve hypothalamic leptin and insulin resistance in high fat diet-induced obesity models in rodents (Al-muzafar and Amin, 2017; Bagarolli et al., 2017; Ji et al., 2018; Cheng and Liu, 2020). Of note, probiotics may prevent AD pathogenesis by improving glucose metabolism. Triple transgenic AD model mice (3xTg-AD) exhibited significant metabolic impairment [increased glycated hemoglobin [HbA1c] in the serum, accumulation of advanced glycation end products (AGE), and impaired glucose uptake due to decreased glucose transporter levels in the brain], and all these parameters were normalized by oral treatment with probiotics (lactic acid bacteria and bifidobacteria) (Bonfili et al., 2020). These data suggest that beneficial gut microbiome can reduce AD pathogenesis via the restoration of metabolic balance (Figure 3).

## Discussion and Conclusion

Metabolic diseases and infection are important risk factors for dementia (Whitmer et al., 2005; Kivipelto et al., 2006; Ashraf et al., 2019; Mejido et al., 2020). Metabolic dysregulation enhances infection susceptibility via immune dysfunction, whereas infection, both in the CNS and periphery, can disrupt metabolic balance (Figure 3). In the CNS, infection-induced damage in the regions associated with metabolic control, such as hypothalamus, can disrupt whole-body energy metabolism. In the periphery, infection-induced immune reactions may persist and disarray immunometabolism, resulting in chronic inflammation and increased BBB permeability (Gustafson et al., 2007; Kanoski et al., 2010; Montagne et al., 2015), which can disrupt the CNS integrity, cognitive ability, and central energy control (Montagne et al., 2015). In addition, infection-induced dysregulation of glucose metabolism can lead to glial activation and neuroinflammation (Huang et al., 2020; Bahar et al., 2021). The vicious cycle may further advance the systemic imbalance, while beneficial gut microbiome can restore the balance of immunometabolism. Furthermore, although evidence is still lacking, infectious agents can potentially disrupt the immunometabolism in the brain by infecting microglia and astrocytes. Thus, multidirectional interactions between metabolic imbalance and infection at cellular and systemic levels likely occur during the development of dementia and neurodegenerative disorders. Combined and multifactorial impacts due to these interactions can further exacerbate brain dysfunction. As the key regulators of energy metabolism and immune responses, insulin and leptin play significant roles in those intricate interactions. It should also be noted that other metabolic hormones and adipokines, such as glucagon and adiponectin, similarly take part in immunometabolism, and dysregulation of these molecules are also implicated in neurodegenerative disorders, including AD (Talbot and Wang, 2014; Grieco et al., 2019; Kim et al., 2020). In addition, growing evidence suggests the central actions of thyroid hormones (THs) (Capelli et al., 2021); THs exert immunomodulatory roles in the brain by inducing microglial phenotypic changes (Mallat et al., 2002) and affecting the expression of chemokines (Davis et al., 2016). Thyroid dysfunctions are possibly associated with AD pathogenesis (Figueroa et al., 2021). Future research may unveil the interactions between infection and these metabolic hormones.

While the potential therapeutic benefit of insulin in treating dementia has been increasingly recognized (Frölich et al., 1998; Kim et al., 2019; Kellar and Craft, 2020), it is relatively unexplored how the modulation of metabolic regulators, including insulin and leptin, can impact infection-induced neurological disorders. Considering the diverse roles for these molecules play in the immunometabolic network, including the CNS immunometabolism (Larabee et al., 2020), it is plausible that infection-induced damages to the brain can be alleviated by modulating these signaling pathways. On the other hand, it is largely unresolved how glia - pathogen interactions can impact immunometabolism in the CNS and what roles leptin and insulin may play in the interactions. How these relationships may relate to the etiology of dementia is also an unanswered question. Further research is warranted to understand the mechanisms underlying the interrelationship between infection, metabolic disorders, and dementia.

## Author Contributions

NS wrote the first draft of the manuscript. KK critically assessed the content. NS and KK jointly finalized the manuscript. Both authors contributed to the article and approved the submitted version.

## Conflict of Interest

The authors declare that the research was conducted in the absence of any commercial or financial relationships that could be construed as a potential conflict of interest.

## Publisher’s Note

All claims expressed in this article are solely those of the authors and do not necessarily represent those of their affiliated organizations, or those of the publisher, the editors and the reviewers. Any product that may be evaluated in this article, or claim that may be made by its manufacturer, is not guaranteed or endorsed by the publisher.
